# The association between food benefit online ordering and redemptions: evidence from the Special Supplemental Nutrition Program for Women, Infants, and Children

**DOI:** 10.1017/S1368980025100931

**Published:** 2025-08-27

**Authors:** Junzhou Zhang, Chuanyi Tang, Kayoung Park, Qi Zhang

**Affiliations:** 1 Department of Marketing, Montclair State University, Montclair, NJ, USA; 2 Department of Marketing, Old Dominion University, Norfolk, VA, USA; 3 Department of Mathematics and Statistics, Old Dominion University, Norfolk, VA, USA; 4 Joint School of Public Health, Macon & Joan Brock Virginia Health Sciences, Old Dominion University, Norfolk, VA, USA

**Keywords:** Special Supplemental Nutrition Program for Women, Infants, and Children, Food benefits redemption, Online grocery shopping

## Abstract

**Objective:**

To examine how the Special Supplemental Nutrition Program for Women, Infants, and Children (WIC) online food benefit ordering could influence WIC benefit redemptions.

**Design:**

A cross-sectional study. We compare the average redemption rates between online ordering early adopters and non-adopters among WIC customers before and after implementing WIC online ordering. A propensity score-weighted difference-in-difference model was used to estimate the coefficients.

**Setting:**

The Oklahoma WIC programme and a grocery store chain in Oklahoma.

**Participants:**

12743 Oklahoma WIC households that had redeemed their food benefits at the grocery store chain in 2020.

**Results:**

WIC online ordering significantly positively affected redemption rates for eight of the fifteen food categories. For example, the difference-in-difference coefficients (*P*–values) of these food categories were cheese or tofu (0·077, <0·01), yogurt (0·092, <0·01), whole milk (0·082, 0·022), low-fat milk (0·060, <0·01), eggs (0·049, 0·033), breakfast cereal (0·085, <0·01) and infant formula (0·073, 0·039). Two food categories with significantly negative difference-in-difference coefficients had relatively lower redemption rates overall: canned fish (Coefficient = –0·209, *P* < 0·01) and infant cereal (Coefficient = –0·138, *P* = 0·015). There were no significant changes in the redemption of fruits and vegetables (Coefficient = 0·031, *P* = 0·121).

**Conclusion:**

Adopting WIC online ordering was positively associated with benefit redemption rates among most food benefit categories. Our findings provide preliminary but important evidence regarding online food benefit redemption among low-income consumers.

Food benefits from the Special Supplemental Nutrition Program for Women, Infants, and Children (WIC), the USA’s third-largest nutrition assistance programme, are essential in promoting low-income participants’ health. Although WIC food benefits are free to participants, under-redemption is prevalent^(1–4)^. For example, Li *et al.* (2021) studied WIC transactions from 2016 to 2018 and found that 18·5 % of the dollar value of WIC food benefits went unredeemed. Literature suggests that factors such as perceived stigmatisation of social assistance programme participants^(5)^, difficulty in memorising and identifying the eligible WIC-authorised brand names or product categories^(6)^, long checkout times^(7)^ and erroneous rejection of eligible transactions in the checkout process^(8)^ contribute to the under-redemption problem.

Policymakers have recently promoted online shopping to improve food access for vulnerable populations^(9)^. Existing studies suggest that online grocery shopping can significantly influence regular consumers’ buying behaviours, such as increasing the effectiveness of product promotions^(10)^, lowering price sensitivity^(11)^, increasing brand loyalty^(12)^ and changing consumers’ perceptions about food items^(13)^. For low-income consumers, online shopping encourages them to plan and budget better^(14,15)^. Therefore, online ordering can improve food benefit redemptions among WIC participants.

Federal regulations require WIC participants to redeem their food benefits before a physical cashier, so the WIC programme does not allow participants to use their electronic benefit transfer cards to order and redeem their food benefits online (7 CFR § 246·12). However, with the waiver approved by the US Department of Agriculture, some WIC agencies and vendors have started piloting ‘online food benefit ordering’ models that allow participants to order their food benefits online but pick up and pay for the order in-store or curbside^(16,17)^. For example, WIC participants can place their food orders on the store app and pick them up in-store or at the curbside later, using their WIC electronic benefit transfer cards for payment.

Similar to how non-WIC customers benefit from online ordering, online food benefit ordering may improve WIC customers’ satisfaction by providing a higher level of convenience, time savings and usefulness^(18,19)^. Moreover, given that WIC shoppers are mainly pregnant females and young mothers with small children, online ordering may be especially beneficial to them. For example, when ordering online, a young mom does not need to select WIC-eligible food items in-store while carrying her baby or trying to manage her children’s whereabouts in the store. Instead, she can order food items online and check out (pay) at the curbside. By doing so, neither she nor her children need to leave the car. WIC participants can also shop online at home without time pressure. In addition, an online ordering system may serve as an antidote to alleviate the embarrassment and stigma often associated with receiving governmental assistance^(20)^. As a final consideration, online ordering makes it easier for WIC participants to identify the correct eligible food items, thus ameliorating transaction rejections, frustrations and embarrassment at the checkout counter and the perceived stigma that participants may experience in the traditional in-store checkout process^(6,21)^. As a result, they are more likely to make full use of their benefits.

Although the WIC programme and food retailers have invested heavily in online benefit ordering, the extent to which such a practice can influence participants’ benefit redemption outcomes remains unknown. The existing studies on this topic mainly used qualitative methods with a small sample^(22,23)^. The findings lack verification from large populations and are not supported by quantitative evidence.

Collaborating with the Oklahoma WIC state agency and a WIC-only grocery store chain that developed and implemented an online ordering system for Oklahoma WIC participants in 2020, this study examined the relationship between the adoption of WIC online ordering and food benefit redemption outcomes. We hypothesised that participants who adopted online ordering would exhibit higher redemption rates across different food categories than those who did not. Online ordering may facilitate the participants’ redemption of standardised food products, such as cereal and milk, for which consumers easily know their quality and usually have their preferred brands and flavours. For other non-standard food categories (particularly fruits and vegetables), consumers may prefer choosing them personally since they are not able to evaluate the quality online. This study helps to fill the knowledge gap in the existing literature on WIC participants’ online grocery shopping practices. It provides crucial and timely information for WIC policymakers seeking to determine the feasibility of expanding WIC online ordering to become standard practice in the WIC programme.

## Methods

### Data

We obtained data from the Oklahoma WIC state agency and a grocery store chain in Oklahoma (the name of the chain is withheld here owing to confidentiality). The Oklahoma WIC agency provided its administrative data from all WIC-authorised stores in Oklahoma for the period from January 2020 to December 2020. The data included the participants’ socio-demographics, their prescribed food benefits (the prescribed dollar or unit value of each food benefit category in a benefit cycle) and their redemption transactions (the redeemed value (in dollars or units) for each food category, the redemption date, and the grocery store of redemption). The store chain has ten WIC-only grocery stores in Oklahoma that offer customers only WIC-eligible foods. It developed the online benefit ordering service for WIC customers in July 2020.

To make the analysis of redemption outcomes comparable before and after implementing WIC online ordering, we kept data only from the retail store chain’s customers who had also redeemed their benefits before the online ordering service was implemented. The grocery store chain’s data provided detailed information about its online and in-store redemption records. We merged the store data with the WIC administrative data. To control for the potential impact of the COVID-19 pandemic, we also retrieved county-level monthly COVID-19 data from the Oklahoma State Department of Health, merging it with the WIC dataset. The final sample included 12 743 WIC households that redeemed their food benefits at the grocery store chain during the study period.

### Measurements

The primary outcome variable was the redemption rate of a household in a benefit cycle, which was defined as the sum of the redeemed amount for a food category divided by the prescribed amount for that food category per household in a benefit cycle. The primary independent variable was the WIC participant’s online ordering adoption status, defined as whether a participating household had used the store’s new WIC online ordering system for at least one redemption trip from July 2020 through December 2020, that is, early adopters *v*. non-adopters. Because WIC online ordering is still at its early diffusion stage and only a small percentage of WIC participants had adopted it at the time of the study^(17)^, some food categories were associated with a very small number of households, causing an insufficient sample size in several food categories to make a valid statistical comparison impossible. Therefore, we removed three prescribed food categories having online orders from fewer than fifty households, including infant meat, exempt infant formula and WIC-eligible nutritionals. Hence, the number of remaining food categories in our final analysis was fifteen. We further grouped these categories into nine broader categories (eggs, breakfast cereal, legumes, canned fish, bread/whole grains, fruits and vegetables, infant food, dairy products and juice). The fruits and vegetables benefits, that is, cash-value benefits, were prescribed in dollar amounts, while all other benefits were prescribed in units such as ounces or gallons.

### Statistical analyses

Descriptive statistics were reported for the socio-demographics of WIC online ordering early adopters *v*. non-adopters, and the differences between these two groups were tested with *χ* ^2^ and Wilcoxon rank-sum tests. Moreover, we used the propensity score-weighted difference-in-difference (DiD) approach to compare redemption rates before and after implementing the WIC online ordering system for different food benefit categories. The purpose of the propensity score approach is to control the potential self-selection bias in adopting WIC online ordering so these two groups can be compared^(24)^. Moreover, since WIC online ordering was piloted in just one grocery store chain, most Oklahoma WIC participants have not had the chance to adopt it, creating unbalanced samples between WIC online-ordering participants and non-online-ordering participants. To address this, we matched households with similar estimated propensity scores to create a balanced sample of households that adopted WIC online ordering and those that did not. The DiD approach has been widely used in social science to simulate an experimental design using observational data by alleviating the confounding effects from exogenous variables over time so the causal effect can be estimated^(25)^. In combination, a propensity score-weighted DiD approach is an integrated approach to further minimising selection bias and accurately estimating the causal effects by utilising the strength of both propensity score and DiD approaches. This method has been applied to study WIC participants’ outcomes in other settings^(24,26)^.

Specifically, we first estimated a propensity score of each household’s adoption status of WIC online ordering (1 for the households that had adopted WIC online ordering and 0 for the others) based on a set of observed variables (e.g., racial or ethnic group, whether the household had an infant/child/woman participant, the number of WIC participants in a household, the overall household size and household income, as shown in Table 1). Next, using the WIC online ordering status variable along with a time binary variable, we created four groups: pre-treatment (WIC online ordering early adopters before the implementation), pre-control (WIC online ordering non-adopters before the implementation), post-treatment (WIC online ordering early adopters after the implementation) and post-control (WIC online ordering non-adopters after the implementation). Further, utilising multinomial logistic regression, we estimated the probabilities of each WIC participant being in one of the four groups. We used these probabilities as sampling weights in estimating the DiD model. The participating households’ socio-demographics were controlled in these analyses.

**Table 1.** Sociodemographic characteristics by WIC online ordering status

	*n* 12 743		Online orders early adopters (*n* 597)		Non-adopters (*n* 12 146)	
Characteristic	*n/mean*	%/SD	*M/mean*	*%/sd*	*M/mean*	*%/sd*
Racial/ethnic group						
Non-Hispanic White	1581	12·4	126	21·1	1455	12·0
Non-Hispanic Black	2013	15·8	76	12·7	1937	15·9
Hispanic	7506	58·9	329	55·1	7177	59·1
Others	1643	12·9	66	11·1	1577	13·0
Did the household have an infant participant?						
Yes	3900	30·6	220	36·9	3680	30·3
No	8843	69·4	377	63·1	8466	69·7
Did the household have a child participant?						
Yes	10 129	79·5	490	82·1	9639	79·4
No	2614	20·5	107	17·9	2507	20·6
Did the household have a woman participant?						
Yes	2943	23·1	162	27·1	2781	22·9
No	9800	76·9	435	72·9	9365	77·1
Number of WIC participants						
= 1	7992	62·7	304	50·9	7688	63·3
= 2	3454	27·1	191	32·0	3263	26·9
≥ 3	1297	10·2	102	17·1	1195	9·8
Household size (number of people)	4·2	1·6	4·1	1·6	4·2	1·6
Income ($)	22 397	1483·6	23 699	14 459·3	22 333	14 846·2

WIC, Special Supplemental Nutrition Program for Women, Infants, and Children.

To control the impact of the COVID-19 pandemic on the redemption, we controlled the number of COVID-19 incidences within the participant’s county of residence in the analyses, a standard approach in the literature^(27–29)^. Since the early days of the COVID-19 pandemic affected the food supply, such as infant formula, we conducted additional robustness checks for the validity of the analyses. The propensity score-weighted DiD approach compares the changes before and after the WIC online ordering implementation between the early adopters and the non-adopters. Therefore, as long as the pandemic affected the two groups equally before the implementation, the DiD model is still a valid comparison approach. The robustness checks confirmed the assumption. We removed all the records when any variables we used in the analysis had missing values. *P* < 0·05 was set as the significance level. Stata 15 was used to conduct the analyses^(30)^. The Institutional Review Board of the corresponding author’s institution conducted an expedited review and approved this study, waiving the need for participants’ consent.

## Results

The socio-demographics of our sample by their WIC online ordering status are presented in Table 1. In the study sample, 58·9 % were Hispanic, followed by non-Hispanic Black (15·8 %), other racial/ethnic groups (12·9 %) and non-Hispanic White (12·4 %). However, 21·1 % of the online-ordering early adopters were non-Hispanic White. In comparison, only 12·7 % were non-Hispanic Black, and 55·1 % were Hispanic, which was significantly different from the non-adopters (*P* < 0·01). Moreover, a significantly higher percentage of online-ordering early adopters had an infant participant (36·9 % *v*. 30·3 %, *P* < 0·01) and a woman participant (27·1 % *v*. 22·9 %, *P* = 0·019), but no significant difference was found among households having a child participant (*P* = 0·120). The online-ordering early adopters also had significantly more WIC participants in their households (*P* < 0·01) but a smaller overall household size (4·1 *v*. 4·2, *P* = 0·010) and a higher income ($23 699 *v*. $22 333, *P* = 0·018).

Table 2 presents the average redemption rates of different food categories between WIC online ordering earlier adopters and non-adopters before and after the store’s online ordering implementation. Before implementation, customers in both groups were not significantly different regarding their average redemption rates for most food categories, with the difference being around one percentage point (*P* > 0·05), except for the 48 fl oz of juice for women participants (5·0 percentage points higher among online ordering early adopters, *P* < 0·01). After the store implemented online ordering, the differences in most categories between online ordering early adopters and non-adopters were enlarged. Several food categories, including fruits and vegetables, infant formula, low-fat milk, cheese or tofu, yogurt and juice (48 f l oz), achieved statistical significance (*P* < 0·05). Among these food categories, the average redemption rates in the WIC online ordering early adopters were all greater than the non-adopters except for low-fat milk (–2·2 percentage points, *P* < 0·01). Notably, the redemption rates of the infant formula were 3·8 percentage points higher among WIC online ordering early adopters (*P* < 0·01). Therefore, we observed a consistent increase in the average redemption rates in multiple food categories after the WIC online ordering implementation.

**Table 2.** Average redemption rates between online WIC ordering early adopters and non-adopters before and after online ordering implementation

	Before WIC online ordering implementation						After WIC online ordering implementation					
	Early adopters (*n* 597)		Non-adopters (*n* 12 146)				Early adopters (*n* 597)		Non-adopters (*n* 12 146)			
Food category	*%*	*sd*	*%*	*sd*	*Difference*	*P*	*%*	*sd*	*%*	*sd*	*Difference*	*P*
Eggs	70·9	42·9	70·4	43·9	0·5	0·870	77·0	40·2	74·9	42·3	2·1	0·088
Breakfast cereal	62·8	45·0	62·9	44·1	−0·1	0·137	60·9	44·0	59·8	45·4	1·1	0·878
Legumes	68·0	44·8	67·5	45·3	0·5	0·764	66·5	45·5	66·7	46·1	−0·2	0·384
Canned fish	63·4	47·7	58·4	48·3	5·0	0·109	61·4	47·5	59·1	48·3	2·3	0·521
Bread/whole grains	66·5	43·9	65·4	45·0	1·1	0·544	64·1	44·6	63·0	45·9	1·1	0·553
Fruits and vegetables	74·4	39·3	74·2	40·5	0·2	0·710	76·6	36·6	76·0	39·1	0·6	<0·01
Infant food												
Infant cereal	59·2	46·8	56·8	47·6	2·4	0·275	53·7	47·9	56·5	47·7	−2·8	0·170
Infant fruits and vegetables	64·8	43·1	61·1	44·3	3·7	0·071	62·6	43·7	60·2	44·9	2·4	0·210
Infant formula	74·5	41·2	75·3	40·5	−0·8	0·920	89·4	27·6	85·6	31·7	3·8	<0·01
Dairy product												
Cheese or tofu	71·6	42·9	70·5	44·1	1·1	0·623	74·4	41·6	72·1	43·6	2·3	0·032
Yogurt	69·6	44·0	68·3	45·2	1·3	0·437	70·9	43·4	68·0	45·4	2·9	<0·01
Whole milk	70·0	39·3	71·0	39·3	−1·0	0·378	71·1	37·9	70·3	39·3	0·8	0·734
Low-fat milk	62·3	38·8	63·1	40·7	−0·8	0·065	62·1	38·3	64·3	40·4	−2·2	<0·01
Juice												
Juice (48 fl oz) for women	63·9	47·0	58·9	48·3	5·0	<0·01	60·7	47·9	57·1	48·7	3·6	0·011
Juice (64 fl oz) for children	73·8	42·1	73·0	42·6	0·8	0·410	74·3	41·3	72·4	43·2	1·9	0·112

WIC, Special Supplemental Nutrition Program for Women, Infants, and Children.

Statistical tests performed: Wilcoxon rank-sum test.

Table 3 presents the results after applying the propensity score-weighted DiD results across different food categories and controlling the socio-demographics and the number of COVID cases. It presents the results among the comparable WIC online ordering early adopters and non-adopters with propensity score matching. In comparison, Table 2 includes the general WIC online ordering non-adopters. The coefficients of the DiD estimator in Table 3 capture the treatment effect of the adoption of WIC online ordering on redemption rates. Out of fifteen food categories, eight coefficients were significantly positive. For example, for all dairy product food categories, the adoption of WIC online ordering was positively associated with redemption rates (Coef. = 0·077, *P* < 0·01 for cheese or tofu; Coef. = 0·092, *P* < 0·01 for yogurt; Coef. = 0·082, *P* = 0·022 for whole milk; Coef. = 0·060, *P* < 0·01 for low-fat milk). For juice categories, juice (64 fl oz) for children (Coef. = 0·049, *P* = 0·035) demonstrated a significantly positive relationship, while the coefficient for juice (48 fl oz) for women was not significant. Additionally, the food categories of eggs (Coef. = 0·049, *P* = 0·033), breakfast cereal (Coef. = 0·085, *P* < 0·01) and infant formula (Coef. = 0·073, *P* = 0·039) showed significantly positive estimates, indicating a positive effect of WIC online ordering adoption on redemption rates. We also found for the food categories of canned fish (Coef. = –0·209, *P* < 0·01) and infant cereal (Coef. = –0·138, *P* = 0·015) that the relationships between WIC online ordering and redemption rates were negative. The relationships for the remaining food categories were not significant.

**Table 3.** Propensity score-weighted difference-in-difference (DiD) analyses of WIC online ordering

	Online ordering implementation periods (before = 0, after = 1)			Online ordering adoption (No = 0, Yes = 1)			DiD (online ordering adoption* after implementation)		
Food category	*Coef.*	*sd*	*P*	*Coef.*	*sd*	*P*	*Coef.*	*sd*	*P*
Eggs	0·028	0·009	<0·01	0·018	0·009	0·038	0·049	0·023	0·033
Breakfast cereal	−0·040	0·009	<0·01	0·018	0·009	0·047	0·085	0·023	<0·01
Legumes	−0·028	0·009	<0·01	0·023	0·009	0·017	0·029	0·025	.253
Canned fish	0·019	0·030	0·522	0·048	0·033	0·142	−0·209	0·071	<0·01
Bread/whole grains	−0·024	0·010	0·013	0·032	0·010	0·001	0·036	0·026	0·166
Fruits and vegetables	0·001	0·008	0·848	0·010	0·008	0·199	0·031	0·020	0·121
Infant food									
Infant cereal	−0·050	0·020	0·012	0·003	0·023	0·903	−0·138	0·056	0·015
Infant fruits and vegetables	−0·038	0·018	0·036	0·029	0·022	0·186	−0·097	0·055	0·079
Infant formula	0·079	0·012	<0·01	−0·026	0·016	0·107	0·073	0·035	0·039
Dairy product									
Cheese or tofu	−0·003	0·009	0·735	0·023	0·009	0·011	0·077	0·022	<0·01
Yogurt	−0·041	0·009	<0·01	0·024	0·009	0·012	0·092	0·025	<0·01
Whole milk	−0·010	0·015	0·497	0·004	0·015	0·797	0·082	0·036	0·022
Low-fat milk	0·004	0·008	0·618	0·013	0·008	0·127	0·060	0·022	<0·01
Juice									
Juice (48 fl oz) for women	−0·030	0·013	0·026	0·058	0·014	<0·001	0·050	0·038	0·192
Juice (64 fl oz) for children	−0·018	0·010	0·067	0·017	0·010	0·092	0·049	0·023	0·035

WIC, Special Supplemental Nutrition Program for Women, Infants, and Children.

Variables controlled: racial groups, household members’ WIC participation status, the number of WIC participants in a household, household size, income, the population of the residential county and the COVID cases in the residential county.

## Discussion

We merged the WIC administrative data and online ordering data from a WIC-only grocery store chain in Oklahoma to examine how the adoption of WIC online ordering was associated with redemption rates across food benefit categories. By employing the propensity score-weighted DiD approach, we made comparable groups of WIC participants to estimate the causal effect of the online ordering option on redemption. In the analysis, we also included the number of COVID cases to control the potential influence of the pandemic. In summary, WIC online ordering significantly positively affected redemption rates for more than half of the food benefit categories, including the most popular WIC food items, such as dairy products, eggs and breakfast cereals. One category without a significant difference was fresh fruits and vegetables. Since the quality of products in this category varies greatly and is difficult to evaluate, WIC participants may prefer to inspect the freshness and ripeness of produce personally. They might appreciate the experience of selecting fresh fruits and vegetables in-store rather than purchasing them online^(16,17,31)^. Consequently, adding an online redemption option did not increase the redemption rate of the cash-value benefits.

This study has important implications for policymakers and WIC-authorised store managers who may consider online ordering for low-income consumers. In general, WIC online ordering was viewed favourably by WIC participants^(15,32)^, but little literature has shown how WIC online ordering improved the participants’ benefit redemption. Our study demonstrated that implementing WIC online ordering is associated with greater benefit redemption. More than half of the food benefit categories were found to experience a significantly higher redemption rate among WIC online-ordering early adopters, even after controlling the effect of the COVID-19 pandemic and the potential self-selection bias in the comparison. This finding is consistent with previous studies indicating that technological innovations may improve redemption rates^(1,4)^. Given the empirical evidence of the overall promising and positive effect of online ordering on most food benefit categories, the states and store chains that have not offered an online ordering system for WIC participants may want to consider the option. WIC agencies may work closely with retailers to expand WIC online ordering to other retail store chains and other states.

We found that the adoption rate of online ordering was highest among non-Hispanic White participants. Measures should be taken to enhance the adoption among other races, including Hispanic and non-Hispanic Black participants. For example, targeted communications via partnerships with local churches, schools, health clinics and nonprofits that serve Hispanic and Black communities should be conducted. Whether this technological innovation increases the disparities in redemption behaviours across races/ethnicities requires more research to ensure equal access to WIC online ordering.

As an empirical study investigating the online ordering behaviours of WIC participants, the present study has a few limitations. First, the online ordering model examined in the study is still in its early deployment stage, lacking the option of online payment and home delivery. The US Department of Agriculture awarded a $2·5 million grant to pilot test WIC online ordering projects^(33)^. In the American Rescue Plan Act of 2021, $390 million was appropriated for WIC system modernisation, programme innovation and enhancing outreach efforts towards existing or potential WIC participants^(34)^. As required by the Consolidated Appropriations Act of 2021, a US Department of Agriculture task force was formed to examine alternative ordering and delivery methods, such as online ordering and payment for WIC food benefits^(35)^. As the federal government further modernises WIC online ordering services, further research should be conducted to explore the impact of online payment and home delivery on redemption rates and investigate diverse redemption behaviours, such as store-switching.

Second, besides the factors that we examined in this study, some other factors (e.g. the store’s efforts in advertising the WIC online ordering system or the participating households’ characteristics, such as vehicle access, health conditions and technology readiness) may also influence WIC participants’ online benefit ordering behaviours and outcomes. Unfortunately, these data are either not available or not accessible. The generalisability of the findings might be limited by the research context: In a WIC-only grocery store chain in Oklahoma, a unique retail format was employed. Moreover, a special period of the COVID-19 pandemic was the study window. Although the unique research context and period might limit the generalisability of our findings, the underlying motivations (e.g. convenience and saving time) and barriers to adopting online ordering (e.g. low technology readiness and preference for in-person shopping) are likely to be consistent across the customers of WIC-only stores. For other general store types, we believe that most of our findings can be applied to WIC benefit redemptions in other contexts. Nonetheless, future research in different retail formats and periods is needed to validate the findings of our study. Although we used the DiD approach to estimate the causal effect from empirical research, this is not the gold standard for natural experiments to examine the causal relationships between online ordering and redemption outcomes.

Even with these limitations, this study is one of the first to examine the online grocery shopping behaviour of WIC participants, who represent a nutritionally vulnerable population of women, infants and children. It complements the literature on WIC online ordering, especially regarding its impact on redemptions. In addition, our study goes beyond the traditional survey and qualitative research approaches in the existing literature by analysing WIC administrative data and online ordering data to understand WIC consumers’ actual online food benefit redemption behaviour.

## Conclusions

WIC modernisation efforts led to the inclusion of the online benefit redemption option, which provides more ways for WIC participants to access the programme’s healthy food benefits. Our data show that this option can improve participants’ benefit redemption rate. The 2017 National Academies of Sciences, Engineering, and Medicine WIC Report emphasises that increasing choice is a goal for serving WIC participants more effectively^(36)^. Our study shows that offering both online ordering and in-store shopping options for the redemption of benefits helps to improve redemption rates across food categories, thus demonstrating that online ordering can contribute to achieving the goals of increasing choice and improving benefit redemption. We call for future studies to examine how this innovation can influence the participants’ redemption behaviours.

## References

[1] Li X , McLaughlin PW , Saitone TL et al. (2021) The magnitude and determinants of partial redemptions of food benefits in the special supplemental nutrition program for women, infants and children (WIC). Am J Health Promot 35, 775–783. 33611926 10.1177/0890117121992307

[2] Payne CR , Niculescu M , Guthrie JF et al. (2018) Can a better understanding of WIC customer experiences increase benefit redemption and help control program food costs? J Hunger Environ Nutr 13, 143–153.

[3] Tang C , Zhang J , Zhang Q et al. (2023) Understanding the chasm in the diffusion of online food benefit ordering: a service ecosystem approach. J Serv Res. Published online: 16 November 2023. doi: 10.1177/10946705231215150.

[4] Zhang Q , Zhang J , Park K et al. (2021) App usage associated with full redemption of WIC food benefits: a propensity score approach. J Nutr Educ Behav 53, 779–786. 34175218 10.1016/j.jneb.2021.03.002

[5] Baumberg B (2016) The stigma of claiming benefits: a quantitative study. J Soc Policy 45, 181–199.

[6] Bertmann FM , Barroso C , Ohri-Vachaspati P et al. (2014) Women, infants, and children cash value voucher (CVV) use in Arizona: a qualitative exploration of barriers and strategies related to fruit and vegetable purchases. J Nutr Educ Behav 46, Suppl 3, S53–S58. 24809997 10.1016/j.jneb.2014.02.003

[7] Chauvenet C , De Marco M , Barnes C et al. (2019) WIC recipients in the retail environment: a qualitative study assessing customer experience and satisfaction. J Acad Nutr Diet 119, 416–424. 30502034 10.1016/j.jand.2018.09.003

[8] Zhang J , Zhang Q , Tang C et al. (2022) The role of generic price look-up code in WIC benefit redemptions. J Public Policy Mark 41, 237–253.

[9] Rogus S , Guthrie JF , Niculescu M et al. (2020) Online grocery shopping knowledge, attitudes, and behaviors among SNAP participants. J Nutr Educ Behav 52, 539–545. 31870741 10.1016/j.jneb.2019.11.015

[10] Zhang J & Krishnamurthi L (2004) Customizing promotions in online stores. Mark Sci 23, 561–578.

[11] Chu J , Chintagunta P & Cebollada J (2008) Research note—a comparison of within-household price sensitivity across online and offline channels. Mark Sci 27, 283–299.

[12] Block LG , Grier SA , Childers TL et al. (2011) From nutrients to nurturance: a conceptual introduction to food well-being. J Public Policy Mark 30, 5–13.

[13] Huyghe E , Verstraeten J , Geuens M et al. (2017) Clicks as a healthy alternative to bricks: how online grocery shopping reduces vice purchases. J Mark Res 54, 61–74.

[14] Richards MR & Sindelar JL (2013) Rewarding healthy food choices in SNAP: behavioral economic applications. Milbank Q 91, 395–412. 23758515 10.1111/milq.12017 PMC3696202

[15] Zimmer M , McElrone M & Anderson Steeves ET (2021) Feasibility and acceptability of a “click & collect” WIC online ordering pilot. J Acad Nutr Diet 121, 2464–2474. 34219049 10.1016/j.jand.2021.05.015

[16] Zimmer MC , Beaird J & Anderson Steeves ET (2021) WIC participants’ perspectives about online ordering and technology in the WIC program. J Nutr Educ Behav 53, 602–607. 33132035 10.1016/j.jneb.2020.10.001

[17] Zhang Q , Park K , Zhang J et al. (2022) The online ordering behaviors among participants in the Oklahoma women, infants, and children program: a cross-sectional analysis. Int J Environ Res Public Health 19, 1805. 35162828 10.3390/ijerph19031805 PMC8835125

[18] Raijas A (2002) The consumer benefits and problems in the electronic grocery store. J Retail Consum Serv 9, 107–113.

[19] Morganosky MA & Cude BJ (2000) Consumer response to online grocery shopping. Int J Retail Distrib Manag 28, 17–26.

[20] Shepherd S & Campbell T (2020) The effect of egocentric taste judgments on stereotyping of welfare recipients and attitudes toward welfare policy. J Public Policy Mark 39, 1–14.

[21] Woelfel ML , Abusabha R , Pruzek R et al. (2004) Barriers to the use of WIC services. J Am Diet Assoc 104, 736–743. 15127057 10.1016/j.jada.2004.02.028

[22] Coffino JA , Han GT & Evans EW (2021) A default option to improve nutrition for adults with low income using a prefilled online grocery shopping cart. J Nutr Educ Behav 53, 759–769. 34509276 10.1016/j.jneb.2021.06.011

[23] Coffino JA , Udo T & Hormes JM (2020) Nudging while online grocery shopping: a randomized feasibility trial to enhance nutrition in individuals with food insecurity. Appetite 152, 104714. 32304731 10.1016/j.appet.2020.104714 PMC7482976

[24] Stuart EA , Huskamp HA , Duckworth K et al. (2014) Using propensity scores in difference-in-differences models to estimate the effects of a policy change. Health Serv Outcomes Res Methodol 14, 166–182. 25530705 10.1007/s10742-014-0123-z PMC4267761

[25] Wing C , Simon K & Bello-Gomez RA (2018) Designing difference in difference studies: best practices for public health policy research. Annu Rev Public Health 39, 453–469. 29328877 10.1146/annurev-publhealth-040617-013507

[26] Li K , Fan JX , Wen M et al. (2022) WIC participation and dietary quality among US children: impact of the 2009 food package revision. J Hunger Environ Nutr 17, 445–459. 36777812 10.1080/19320248.2022.2070444 PMC9910511

[27] Dasgupta S , Kassem AM , Sunshine G et al. (2021) Differences in rapid increases in county-level COVID-19 incidence by implementation of statewide closures and mask mandates—United States, June 1–September 30, 2020. Ann Epidemiol 57, 46–53. 33596446 10.1016/j.annepidem.2021.02.006 PMC7882220

[28] Islam SJ , Nayak A , Hu Y et al. (2021) Temporal trends in the association of social vulnerability and race/ethnicity with county-level COVID-19 incidence and outcomes in the USA: an ecological analysis. BMJ Open 11, e048086. 10.1136/bmjopen-2020-048086 PMC8300549 34301657

[29] Wang L , Zhang S , Yang Z et al. (2021) What county-level factors influence COVID-19 incidence in the United States? Findings from the first wave of the pandemic. Cities 118, 103396. 34334868 10.1016/j.cities.2021.103396 PMC8316070

[30] StataCorp L (2015) Stata Statistical Software: Release 15. College Station, TX: StataCorp, LLC.

[31] Kühn F , Lichters M & Krey N (2020) The touchy issue of produce: need for touch in online grocery retailing. J Bus Res 117, 244–255.

[32] Pitts SBJ , Ng SW , Blitstein JL et al. (2020) Perceived advantages and disadvantages of online grocery shopping among special supplemental nutrition program for women, infants, and children (WIC) participants in Eastern North Carolina. Curr Dev Nutr 4, nzaa076. 32399508 10.1093/cdn/nzaa076 PMC7204786

[33] US Department of Agriculture (2020) WIC online ordering grant. Available at https://www.fns.usda.gov/grant/wic-online-ordering (accessed March 2024).

[34] H.R. 1319–117th Congress (2021) American Rescue Plan Act of 2021. Available at https://www.congress.gov/bill/117th-congress/house-bill/1319 (accessed July 2024).

[35] US Department of Agriculture (2021) Taskforce on supplemental food delivery in the WIC program: recommendations report. Available at https://www.fns.usda.gov/wic/food-delivery-task-force-recommendations-report (accessed November 2023).

[36] National Academies of Sciences, Engineering, and Medicine (2017) Review of WIC Food Packages: Improving Balance and Choice: Final Report. Washington, DC: The National Academies Press. 28605175

